# Effects of Modifying Thioflavin T at the *N*^3^-Position on Its G4 Binding and Fluorescence Emission

**DOI:** 10.3390/molecules25214936

**Published:** 2020-10-26

**Authors:** Yuka Kataoka, Hiroto Fujita, Tamaki Endoh, Naoki Sugimoto, Masayasu Kuwahara

**Affiliations:** 1 Graduate School of Integrated Basic Sciences, Nihon University, 3-25-40 Sakurajosui, Setagaya-ku, Tokyo 156-8550, Japan; kataoka.yuka@nihon-u.ac.jp (Y.K.); fujita.hiroto@nihon-u.ac.jp (H.F.); 2 Frontier Institute for Biomolecular Engineering Research (FIBER), Konan University, 7-1-20, Minatojima-Minamimachi, Chuo-ku, Kobe 650-0047, Japan; t-endoh@konan-u.ac.jp (T.E.); sugimoto@konan-u.ac.jp (N.S.); 3 Graduate School of Frontiers of Innovative Research in Science and Technology (FIRST), Konan University, 7-1-20 Minatojima-Minamimachi, Chuo-ku, Kobe 650-0047, Japan

**Keywords:** G-quadruplex, G4-binder, G4-probe, thioflavin T derivative

## Abstract

We previously synthesized thioflavin T (ThT) with a hydroxyethyl group introduced at the *N*^3^-position (ThT-HE), which binds predominantly to the parallel G-quadruplex (G4) structure found in c-Myc and emits strong fluorescence. In this study, to investigate the effects of introduced substituents on G4 binding and fluorescence emission, a ThT derivative in which the hydroxyl group of ThT-HE was replaced with an amino group (ThT-AE) was synthesized for the first time. Furthermore, three other *N*^3^-modified ThT derivatives (ThT-OE2, ThT-SP, and ThT-OE11) having different substituent structures were synthesized by the N-acylation of the terminal amino group of ThT-AE, and their G4-binding and emission properties were investigated. The results showed that, although ThT-AE shows binding selectivity depending on the type of G4, its emission intensity is significantly decreased as compared to that of ThT-HE. However, ThT-OE11, which features an 11-unit oxyethylene chain attached to the terminal amino group of ThT-AE, regained about one-half of the emission intensity of ThT-HE while retaining selectivity for G4s. Accordingly, ThT-OE11 may be used as a key intermediate for synthesizing the conjugates of G4 binders and probes.

## 1. Introduction

**Figure 1:**
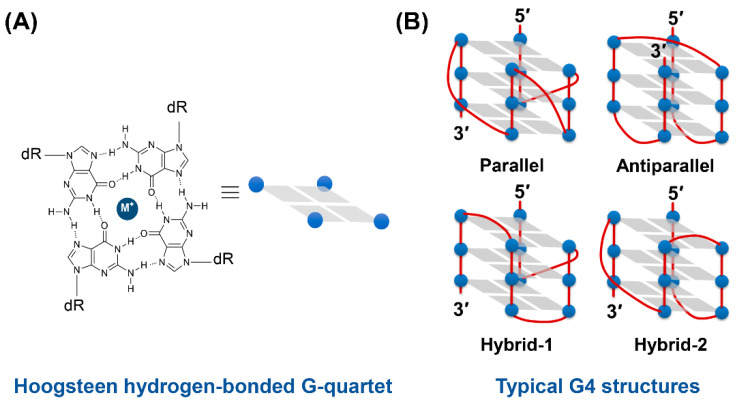
(**A**) Chemical structure of a G-quartet (M^+^ indicates a monovalent cation) and (**B**) illustration of typical G4 structures.

G-quadruplex structures, known as non-canonical nucleic acid structures, are likely to occur in sequences rich in guanine. G-quadruplex (G4) structures are formed by four Hoogsteen-paired coplanar guanines, which are called a G-quartet, via stacking interactions. G4 structures are stabilized by the coordination of cations such as sodium or potassium ions in the center of the G-quartet. Typical examples of G4 structures are parallel, antiparallel hybrid-1, and hybrid-2 types (Figure 1) [1,2]. These G4 structures are present mainly in the telomere and promoter regions, and it has been confirmed that the induction of the structure stabilizes chromosomes, controls replication on–off, and protects the telomere region from telomerase. In the promoter region, it has been reported that RNA polymerase binding is inhibited by inducing a G4 structure, as transcription itself is inhibited, and transcription progress is halted in the middle, but the actual roles and functional mechanisms of G4 structures remain unclear [3,4]. Therefore, there is significant research effort focused on the creation of G4 binders and probes (antibodies, small molecular ligands, nucleic acid aptamers, etc.) as a means to elucidate the biological significance of G4 structures and develop fluorescent probes and drug candidates for gene expression regulation.

Several ligands and fluorescent probes that stain G4 structures have been reported [5,6,7,8,9]. Among them, thioflavin T (ThT) has attracted significant attention since Mohanty et al. reported that it can bind G4 structures and emit strong fluorescence with low background emission (i.e., the dye does not emit when it is only bound to dsDNA or ssDNA) [10].

**Figure 2:**
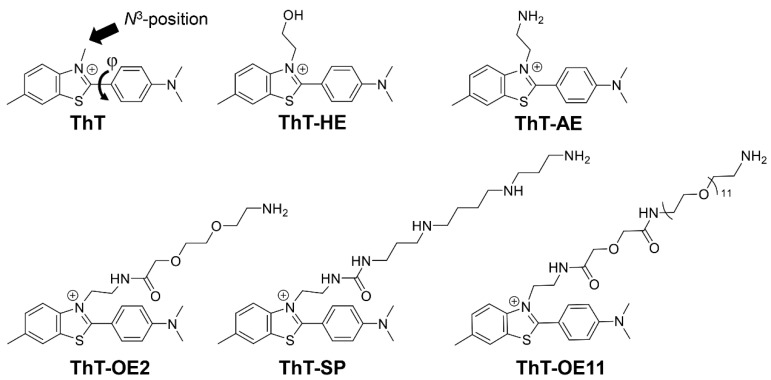
Chemical structures of ThT, ThT-HE, ThT-AE, ThT-OE2, ThT-SP, and ThT-OE11.

The fluorescence intensity of ThT is known to depend greatly on the dihedral angle (φ) between its benzothiazole and dimethylaminobenzen rings [11,12,13] (Figure 2). The ground and excited states of free ThT in solution have φ values of 37° and 90°, respectively, at its lowest potential energy. The excited state of free ThT (φ = 90°) does not emit fluorescence (quantum yield: Φ < 0.001) owing to the radiationless deactivation caused by non-radiative twisted internal charge transfer [14,15]. However, when ThT binds to a target, φ is restricted to 20°–40° in the excited state and fluorescence emission derived from the locally excited state can be observed (Φ = 0.3–0.8) [16]. Therefore, high contrast images with low background fluorescence can be obtained using ThT without removing excess probe molecules.

Since the *N*^3^-position of ThT is closest to the C–C single bond, i.e., the axis of rotation between the rings, it is conceivable that the degree of steric hindrance at this position has a large effect on the degree of rotational freedom. Therefore, in a previous study, we focused on the *N*^3^-position of ThT and introduced a hydroxyethyl group to develop ThT-HE. We then demonstrated that ThT-HE binds to parallel G4 in particular and exhibits strong fluorescence while maintaining the low background emission properties of ThT [17]. In addition, we reported the application of ThT-HE to G4 detection systems [18,19,20]. Since then, ThT with an ethyl group introduced at the *N*^3^-position (ThT-E) has been reported by Guan et al. [21], but the derivatization of ThT at the *N*^3^-position has not seen significant progress otherwise. 

In this study, we synthesized a new thioflavin T derivative in which the hydroxyl group of ThT-HE was replaced with an amino group (ThT-AE). Furthermore, three types of *N*^3^-modified ThT derivatives in which two-unit oxyethylene and spermine chains and an 11-unit oxyethylene chain were introduced by the N-acylation of the terminal amino group were synthesized (Figure 2). By comparing and verifying the binding of these derivatives to G4 and their resultant fluorescence emissions, the effects of the substituents introduced at the *N*^3^-position on G4 binding and fluorescence emission was investigated.

## 2. Results

### 2.1. Fluorescence Spectral Analysis of ThT, ThT-HE, and ThT-AE

**Figure 3:**
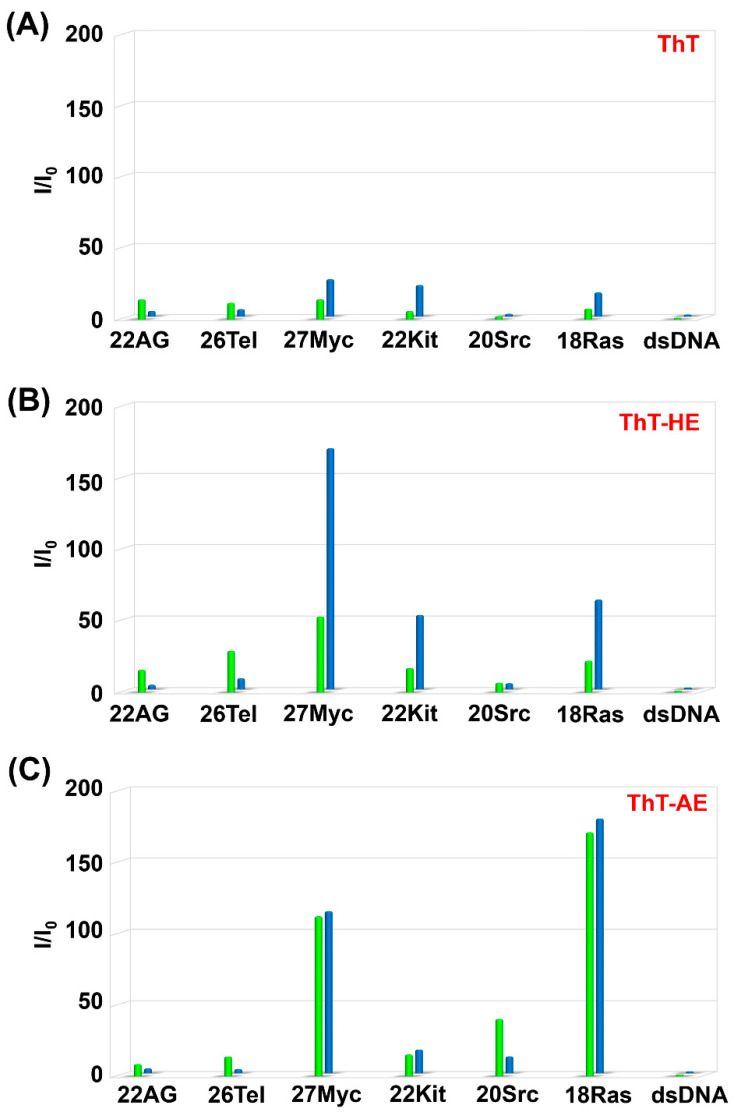
Relative fluorescence intensities (I/I_0_) of (**A**) ThT, (**B**) ThT-HE, and (**C**) ThT-AE with each oligonucleotide in PBS140KM (green) and PBS153NM (blue). Here, I/I_0_ is the ratio of the intensities of the fluorescence emitted upon the binding of the dye (3 μM) to the oligonucleotide (15 μM) and dsDNA (15 μM; duplex concentration), respectively, in the same buffer. All samples were excited at 415 nm and the emissions were monitored at 485 nm.

In order to investigate the fluorescence properties of ThT, ThT-HE, and ThT-AE in the presence of various G4s, fluorescence titration analysis was performed and relative fluorescence intensities were calculated (Figure 3, Table S1). In the fluorescence titrations, we used PBS140KM buffer containing 140 mM K^+^, which simulates intracellular conditions, and PBS153NM buffer containing 153 mM Na^+^, which simulates extracellular conditions. We analyzed six types of human-derived G4 sequences with various topologies under both buffer conditions. dsDNA was used as a background control as it cannot form G4 structures (Table 1). The name of the G4 sequence was defined by the length of the sequence and its origin, and these topologies were defined by the CD spectra in [17] (Figure S1). 

Comparing the relative fluorescence intensities of ThT-HE and ThT-AE with that of ThT, it is clear that, under both Na^+^ and K^+^ buffer conditions, ThT-HE and ThT-AE have improved selectivity for parallel G4s such as 27Myc, 22Kit, 20Src, and 18Ras (Figure 3). Furthermore, the fluorescence intensity of ThT-HE with parallel G4 in Na^+^ buffer is 4.0–170-fold higher than that of dsDNA, but in K^+^ buffer it is significantly decreased (6.6–53-fold).

Conversely, the relative fluorescence intensities of ThT-AE with parallel G4s are 12–180-fold higher in Na^+^ buffer and 15–170-fold higher in K^+^ buffer than those of dsDNA under corresponding conditions. Thus, ThT-AE has the same degree of selectivity for parallel G4 under both conditions. Therefore, the G4 selectivity of ThT-AE in K^+^ buffer is improved compared to that of ThT-HE under similar conditions. 

**Figure 4:**
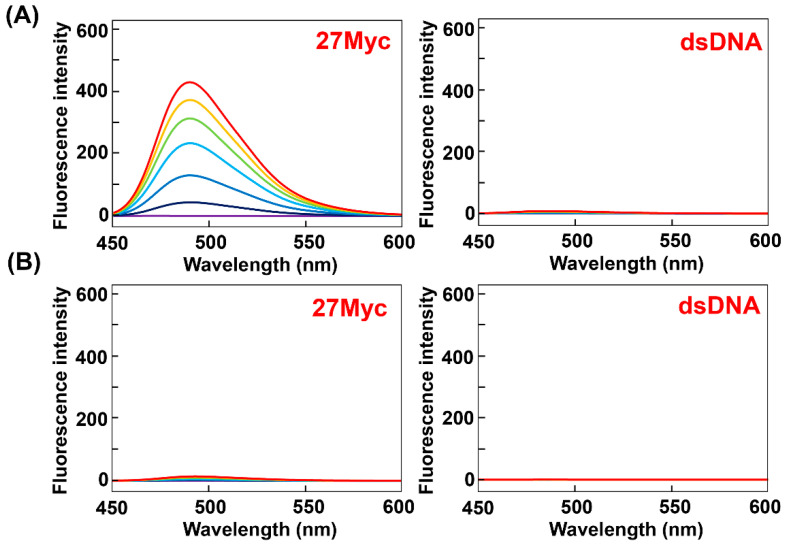
Fluorescence spectra of (**A**) ThT-HE and (**B**) ThT-AE (3 μM) in PBS140KM with increasing concentrations of 27Myc (left side) and dsDNA (right side) (0, 1, 3, 6, 9, 12, and 15 μM). Oligonucleotide concentration: 0 μM (purple), 1 μM (navy blue), 3 μM (blue), 6 μM (light blue), 9 μM (green), 12 μM (yellow), and 15 μM (red).

Furthermore, comparing the fluorescence spectra of ThT-HE and ThT-AE in K^+^ buffer, the fluorescence intensity of ThT-AE with dsDNA is 75-fold lower than that of ThT-HE, but the fluorescence intensity of ThT-AE with 27Myc is 33-fold lower than that of ThT-HE. Therefore, the selectivity of ThT-AE for G4 is relatively high, but the fluorescence intensity is decreased significantly (Figure 4 and Figure S2).

### 2.2. Fluorescence Spectral Analysis of ThT-OE2, ThT-SP, and ThT-OE11

**Figure 5:**
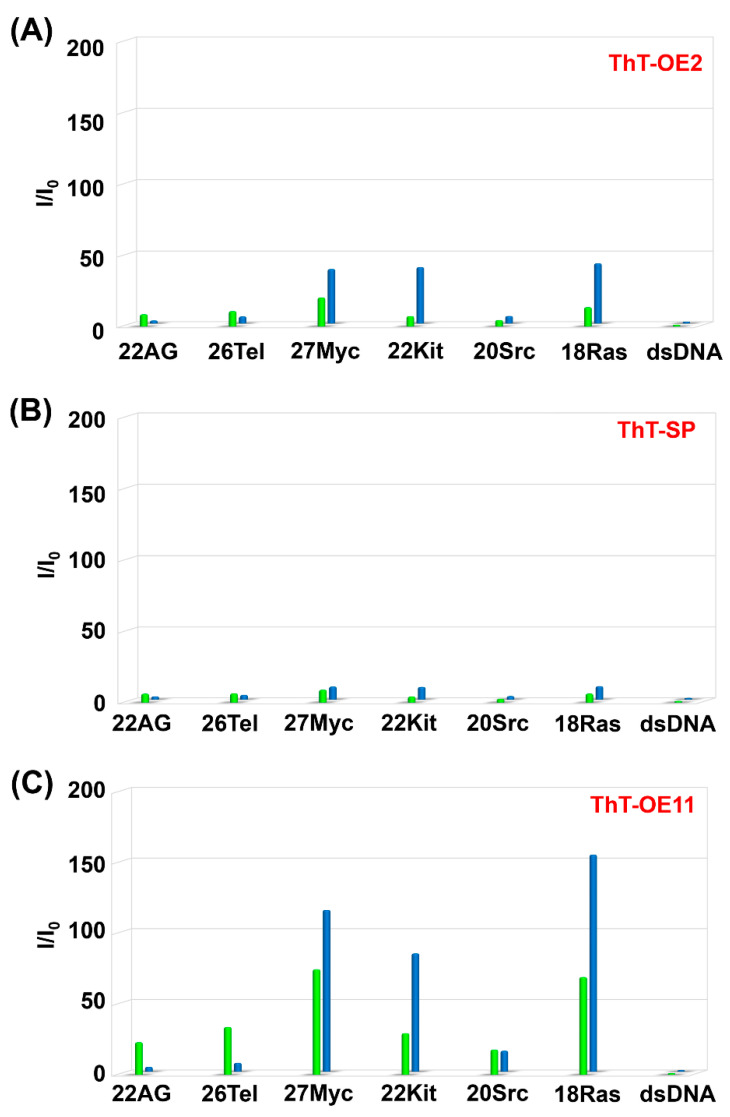
Relative fluorescence intensities (I/I_0_) for (**A**) ThT-OE2, (**B**) ThT-SP, and (**C**) ThT-OE11 with each oligonucleotide in PBS140KM (green) and PBS153NM (blue). Here, I/I_0_ is the ratio of the fluorescence intensities emitted upon the binding of the dye (3 μM) to the oligonucleotide (15 μM) and dsDNA (15 μM; duplex concentration), respectively, in the same buffer. All samples were excited at 415 nm and the emissions were monitored at 485 nm.

The fluorescence properties of ThT-OE2, ThT-SP, and ThT-OE11 in the presence of various G4s were investigated using Na^+^ and K^+^ buffers. Comparing the relative fluorescence intensities in Na^+^ and K^+^ buffers indicates that ThT-OE2 and ThT-SP exhibit poor selectivity for G4 topological structures (Figure 5 and Table S1). Furthermore, the relative fluorescence intensity of ThT-OE11 for parallel G4s is 15–150-fold higher in Na^+^ buffer and 18–75-fold higher in K^+^ buffer. These results show that the selectivity of ThT for parallel G4 is maintained when a long linker chain was introduced at the *N*^3^-position. Furthermore, the selectivity of ThT-OE11 in K^+^ buffer for parallel G4 is around 3.1-fold higher than that of ThT-HE, suggesting an improvement in the selectivity of ThT-OE11 for a parallel G4 in K^+^ buffer. 

**Figure 6:**
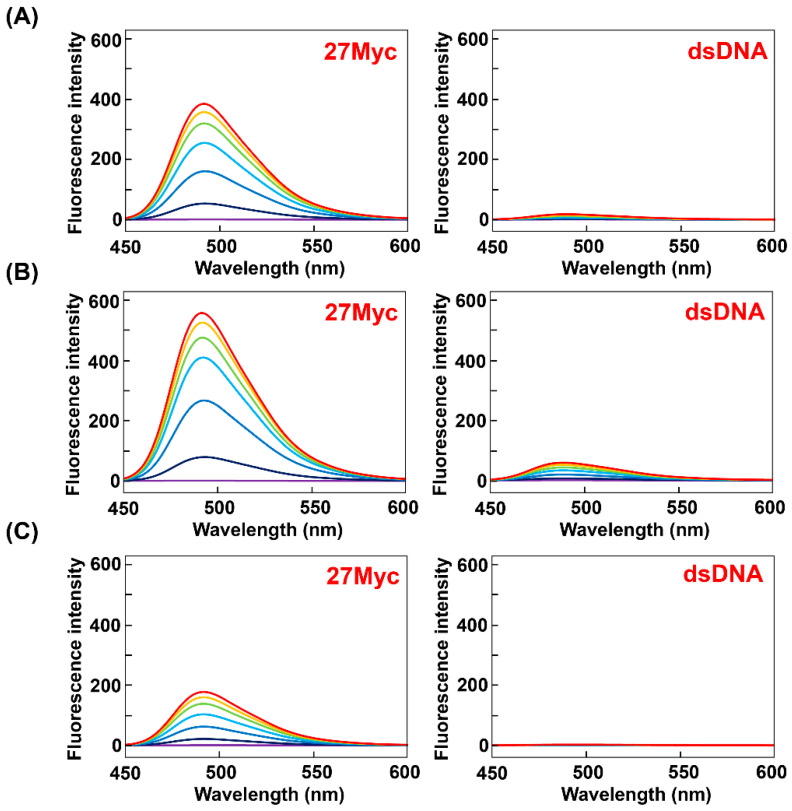
Fluorescence spectra of (**A**) ThT-OE2, (**B**) ThT-SP, and (**C**) ThT-OE11 (3 μM) in PBS140KM with increasing concentrations of 27Myc (left side) and dsDNA (right side) (0, 1, 3, 6, 9, 12, and 15 μM). Oligonucleotide concentration: 0 μM (purple), 1 μM (navy blue), 3 μM (blue), 6 μM (light blue), 9 μM (green), 12 μM (yellow), and 15 μM (red).

A comparison of the fluorescence spectra obtained in K^+^ buffer reveals that the fluorescence intensities of ThT-OE2, ThT-SP, and ThT-OE11 with 27Myc are 27-, 40-, and 13-fold higher than that of ThT-AE (Figure 6, Figures S3 and S4). Therefore, the fluorescence intensity is restored by introducing substituent groups to ThT-AE by N-acylation of the terminal amino group.

Furthermore, although the fluorescence intensity of ThT-OE11 with 27Myc is 2.6-fold lower than that of ThT-HE, the fluorescence intensity of ThT-OE11 with dsDNA is 3.7-fold lower, indicating that ThT-OE11 has a lower background fluorescence than that of ThT-HE.

### 2.3. Binding Affinity and UV–Vis Spectral Analysis 

The apparent dissociation constants (*K*_d_) of these probes with G4s were determined from the fluorescence titration curves obtained above (Table 2 and Figure S5). The *K*_d_ values of ThT-HE, ThT-OE2, and ThT-OE11 for parallel G4s, in particular 27Myc and 18Ras, in both K^+^ buffer and Na^+^ buffer, are several μM to several tens of μM, and there is no difference in the values for the different buffers. Furthermore, the binding affinities for dsDNA are low and fluorescence emission is barely observed because the molecules do not bind to dsDNA. ThT-AE has a low binding affinity for dsDNA under both buffer conditions, suggesting that the fluorescence intensity may be low due to the low binding affinity. Conversely, ThT-SP shows high binding affinity not only for the G4 sequence but also for dsDNA, further indicating its high background fluorescence (Figure 6).

**Figure 7:**
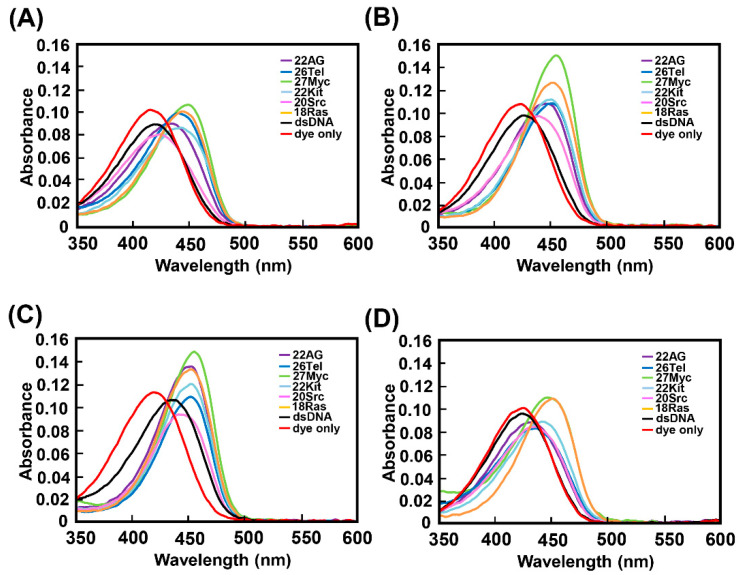
UV-Vis absorption spectra of (**A**) ThT-HE, (**B**) ThT-OE2, (**C**) ThT-SP, and (**D**) ThT-OE11 (3 μM) with or without oligonucleotides (15 μM) in PBS140KM. Oligonucleotides: 22AG (purple), 26Tel (blue), 27Myc (yellow green), 22Kit (light blue), 20Src (pink), 18Ras (yellow), dsDNA (black), and dye only (red).

Figure 7 shows the UV–Vis spectra of ThT-HE, ThT-OE2, ThT-SP, and ThT-OE11 with high binding affinities for parallel G4s in K^+^ buffer. The UV–Vis spectra of ThT-HE, ThT-OE2, and ThT-OE11 (Figure 7A,B,D) are red-shifted by approximately 20 nm due to binding to parallel G4s compared to those of the dye only. Furthermore, the UV–Vis spectra of ThT-SP with the G4s are red-shifted by approximately 30 nm compared to that of ThT-SP alone due to G4 binding, and the spectrum of ThT-SP with dsDNA is also red-shifted by approximately 20 nm due to its binding with dsDNA (Figure 7C). Therefore, the degree of the red shift depends on the degree of change in fluorescence intensity and the binding affinity, suggesting that the probes are stabilized by binding to the target and π-conjugation spread over the ThT framework. These data support the results of fluorescence titration.

## 3. Discussion

The above results demonstrate that ThT-AE has high selectivity for parallel G4s, but the fluorescence intensity is significantly reduced compared with that of ThT-HE. Conversely, ThT-OE11, in which an 11-unit oxyethylene chain is introduced, exhibits high selectivity for parallel G4s and low background fluorescence while maintaining fluorescence intensity when binding to parallel G4s. This may be because the introduced polyethylene glycol linker stabilizes the structure of G4 [22,23]. Thus, the difference between the background fluorescence intensity and that when bound to G4 is larger for ThT-OE11 than that for ThT-OE2, in which a 2-unit oxyethylene chain is introduced, due to the stabilization of the G4 structure. 

Furthermore, the introduction of a spermine moiety (i.e., ThT-SP) [24,25] which binds to DNA or RNA with canonical nucleic acid structures, decreasing G4 selectivity. Therefore, this result shows that the specificity of *N*^3^-modified ThT derivatives can be modulated by chemical moieties to be introduced. In other words, it is possible to develop G4-binders with enhanced specificity for 27Myc by conjugating Phen-Pr, MYRA-A, and NSC308838, which are G4 binders that specifically bind to 27Myc and to ThT-OE11 [26,27]. It may also be possible to develop a G4 probe that enables the ratiometric fluorescence detection of parallel G4s by, for example, conjugating carbon quantum dots to ThT-OE11 [28].

## 4. Materials and Methods

### 4.1. Materials

Oligonucleotides were purchased from Japan Bio Services Co., Ltd. (Saitama, Japan) and GeneDesign Inc. (Osaka, Japan) (Table 1). These were derived from human genes [29,30,31,32,33]. Specifically, telomeric regions (22AG and 26Tel), the nuclease hypersensitivity element region of the c-Myc P1 promoter (27Myc), 87 nucleotides upstream of the initial transcription site of proto-oncogene encoding for a tyrosine kinase (22Kit), proto-oncogene encoding for a nonreceptor tyrosine kinase (20Src), and the 5′-untranslated region of the human NRAS proto-oncogene transcript (18Ras). dsDNA was used as a reference as in previous works [10,17].

To analyze the effects of the buffer condition on fluorescence emission, two buffer solutions were used: PBS140KM (80 mM HPO_4_^2−^, 2.5 mM SO_4_^2−^, 140 mM K^+^, 10 mM Na^+^, 2.5 mM Mg^2+^; pH 7.4), and PBS153NM (10 mM HPO_4_^2−^, 146 mM Cl^−^, 153 mM Na^+^, 2.7 mM K^+^, 2.5 mM Mg^2+^; pH 7.4). PBS140KM (K^+^ buffer) is a mimic of intracellular ionic components, and PBS153NM (Na^+^ buffer) is a typical phosphate saline. All other reagents were of research grade.

### 4.2. Chemical Syntheses of ThT Derivatives

The synthesis of ThT-HE is described in [17] and those of ThT-AE and ThT-OE11 are described in [18]. The syntheses of ThT-OE2 and ThT-SP are described in the supplementary materials.

### 4.3. Fluorescence Spectral Analysis

Oligonucleotides were dissolved in PBS140KM or PBS153NM at 21 μM (a 42 μM mixture of two ssDNAs at a ratio of 1:1 was used as a dsDNA) and refolded by denaturing at 95 °C (or at 40 °C for 18 Ras) for 0.5 min followed by cooling to 25 °C at a rate of 0.5 °C/min using a thermal cycler (WK-0518, Osaka, Japan). These solutions were diluted to appropriate oligonucleotide concentrations, and aliquots (50 μL each) were incubated at 25 °C for 30 min and then mixed with 20 μL solutions of the dye under investigation (ThT, ThT-HE, ThT-AE, ThT-OE2, ThT-SP, or ThT-OE11; 10.5 μM) in PBS140KM or PBS153NM. These mixtures were incubated at 25 °C for 30 min. Emission spectra were obtained by excitation at 415 nm and monitoring fluorescence between 450 and 600 nm (Figure 4 and Figure 6, Figures S2−S4). The fluorescence intensity of the dye (ThT, ThT-HE, ThT-AE, ThT-OE2, ThT-SP, or ThT-OE11; 3 μM) mixed with dsDNA (15 μM) in each buffer solution (defined as I_0_), which generally yielded the minimum fluorescence enhancement and can be defined as background fluorescence, was set to 1. Then, the relative fluorescence intensities of the corresponding dyes in the respective buffer solutions (I/I_0_) were determined (Figure 3 and Figure 5, Table S1). The *K*_d_ values were determined from the titration curves in Figure S5 and are listed in Table 2.

## 5. Conclusions

We succeeded in synthesizing ThT-OE11, which exhibited low background fluorescence and high fluorescence emission intensity and selectivity for parallel G4s, for the first time.

In the future, we hope that ThT-OE11 will be used as an important intermediate in the development of better G4 probes as well as G4 binders, which can be applied as gene expression regulators.

## Figures and Tables

**Table 1:** Synthetic oligonucleotides used in this study.

Oligonucleotide	Sequence	Conformation	
		Na^+^	K^+^
22AG	5’-AGG GTT AGG GTT AGG GTT AGG G-3’	Antiparallel	Hybrid-1
26Tel	5’-TTA GGG TTA GGG TTA GGG TTA GGG TT-3’	Antiparallel	Hybrid-2
27Myc	5’-TGG GGA GGG TGG GGA GGG TGG GGA AGG-3’	Parallel	Parallel
22Kit	5’-AGG GAG GGC GCT GGG AGG AGG G-3’	Parallel	Parallel
20Src	5’-GGG CGG CGG GCT GGG CGG GG-3’	Parallel	Parallel
18Ras	5’-GGG AGG GGC GGG UCU GGG-3’	Parallel	Parallel
dsDNA	5’-GGG TTA CTA CGA ACT GG-3’	Duplex	Duplex
	3’-CCC AAT GAT GCT TGA CC-5’		

**Table 2:** Binding affinities of ThT and its analogs for the target oligonucleotides.

Dye	Buffer	*K*_d_ (μM) ^a^						
		Oligonucleotide						
		22AG	26Tel	27Myc	22Kit	20Src	18Ras	dsDNA
ThT	PBS140KM	5.0 ± 0.5	4.0 ± 0.4	6.9 ± 0.8	9.9 ± 1.2	n.d.	19.0 ± 2.9	n.d.
	PBS153NM	n.d.	29.5 ± 5.6	13.5 ± 5.1	4.8 ± 0.8	n.d.	15.3 ± 1.6	n.d.
ThT-HE	PBS140KM	20.1 ± 1.5	11.1 ± 1.5	19.9 ± 0.9	34.9 ± 6.6	n.d.	25.0 ± 3.1	n.d.
	PBS153NM	n.d.	n.d.	14.1 ± 1.5	12.3 ± 1.8	n.d.	28.1 ± 4.0	n.d.
ThT-AE	PBS140KM	n.d.	9.7 ± 5.5	n.d.	8.9 ± 3.5	n.d.	n.d.	n.d.
	PBS153NM	23.6 ± 9.1	n.d.	n.d.	n.d.	n.d.	n.d.	n.d.
ThT-OE2	PBS140KM	11.8 ± 1.4	5.3 ± 0.4	8.7 ± 0.7	20.5 ± 1.4	38.6 ± 5.9	11.8 ± 0.6	n.d.
	PBS153NM	n.d.	34.3 ± 4.0	10.8 ± 1.3	n.d.	n.d.	15.5 ± 1.7	n.d.
ThT-SP	PBS140KM	5.4 ± 1.0	2.4 ± 0.4	6.8 ± 0.9	6.8 ± 0.6	18.8 ± 2.4	12.8 ± 1.9	16.0 ± 1.0
	PBS153NM	8.2 ± 0.8	4.6 ± 0.3	5.3 ± 0.8	7.4 ± 3.9	4.1 ± 0.5	14.7 ± 3.2	9.4 ± 1.0
ThT-OE11	PBS140KM	n.d.	23.9 ± 3.1	13.4 ± 0.8	36.6 ± 4.4	20.2 ± 2.3	15.3 ± 1.9	n.d.
	PBS153NM	20.3 ± 9.9	n.d.	29.1 ± 2.5	n.d.	n.d.	16.3 ± 1.8	n.d.

^a^ Apparent *K*_d_ values were determined by fluorescence titration. Examples of titration curves are shown in Figure S5. Here, n.d. denotes that accurate values could not be determined under the experimental conditions; apparent *K*_d_ values were assumed to be >40 μM.

## Supplementary Materials

The following are available online, Figure S1. CD spectra for 22AG, 26Tel, 27Myc, 22Kit, 20Src, and 18Ras, Figure S2: Fluorescence spectra of ThT-HE and ThT-AE in PBS140KM, Figure S3: Fluorescence spectra of ThT-OE11 in PBS140KM with 22AG, 26Tel, 22Kit, 20Src and 18Ras, Figure S4: Fluorescence spectra of ThT-OE2, ThT-SP, and ThT-OE11 in PBS140KM, Figure S5: Fluorescence titration curves of ThT, ThT-HE, ThT-AE, ThT-OE2, ThT-SP, and ThT-OE11, Table S1: Fluorescence intensities of ThT, ThT-HE, ThT-AE, ThT-OE2, ThT-SP and ThT-OE11, Scheme S1: Synthetic routes for *N*^3^-modified thioflavin T derivatives (ThT-OE2 and ThT-SP).
