# Supplementary material for: Effects of Modifying Thioflavin T at the N3-Position on Its G4 Binding and Fluorescence Emission
Source: Molecules. 2020 Oct 26;25(21):4936. doi: 10.3390/molecules25214936 (PMC7663003; doi:10.3390/molecules25214936)
Supplement: Supplementary file 1 [file molecules-25-04936-s001.pdf]

Supporting Information

Effects of modifying thioflavin T at the N^3-position on its G4 binding and fluorescence emission

Yuka Kataoka,[1] Hiroto Fujita,[1] Tamaki Endoh,[2] Naoki Sugimoto,[2,3] Masayasu Kuwahara[*,1]

[1]Graduate School of Integrated Basic Sciences, Nihon University, 3-25-40 Sakurajosui, Setagaya-ku, Tokyo 156-8550, Japan

[2]Frontier Institute for Biomolecular Engineering Research (FIBER), Konan University, 7-1-20, Minatojima-Minamimachi, Chuo-ku, Kobe 650-0047, Japan

[3]Graduate School of Frontiers of Innovative Research in Science and Technology (FIRST), Konan University, 7-1-20 Minatojima-Minamimachi, Chuo-ku, Kobe 650-0047, Japan

*To whom correspondence should be addressed. E-mail: mkuwa@chs.nihon-u.ac.jp

Experimental Details

1. General

NMR spectra were measured using NMR instruments (JNM-ECS 400 and JNM-ECA 600, JEOL Ltd., Tokyo, Japan). ESI-mass spectra were recorded by an API 2000 mass spectrometer (Applied Biosystems Inc., Tokyo, Japan). A spectrophotometer (UV-2700, Shimadzu Corporation, Kyoto, Japan) was used to record UV spectra, while a spectrofluorophotometer (LS-55, Perkin Elmer Japan Co., Ltd., Kanagawa, Japan) was used to measure fluorescence spectra. Reverse-phase high-performance liquid chromatography (HPLC) analyses and separations were performed using a HPLC system (JASCO Corporation, Tokyo, Japan).

2. Materials

Sodium hydride was purchased from Nacalai Tesque, Inc. (Kyoto, Japan). 2-(2-Aminoethoxy)ethanol, ethyl trifluoroacetate (TFAEE), methyl bromoacetate, sodium borohydride ($NaBH_4$) and trifluoroacetic acid (TFA) were purchased from Tokyo Chemical Industry Co., Ltd. (Tokyo, Japan). Silica gel 60 were purchased from Kanto Chemical Co., Inc. (Tokyo, Japan). Acetonitrile (MeCN) for liquid chromatography LiChrosolv®, Methanol (MeOH) for liquid chromatography LiChrosolv® and Sodium azide were purchaced from Merk Millipore (Darmstadt, Germany). Acetonitrile (MeCN), dichloromethane (CH_2Cl_2), diethyl ether, *N,N*-dimethylformamide (DMF), 1,4-dioxane, disodium hydrogen phosphate (Na_2HPO_4), ethanol (EtOH), ethyl acetate, ethylene glycol, hydrochloric acid (HCl aq.), magnesium chloride hexahydrate, methanol (MeOH), potassium chloride, potassium dihydrogen phosphate (KH_2PO_4), potassium hydrogen sulfate ($KHSO_4$), sodium chloride (NaCl), sodium dihydrogen phosphate dihydrate ($NaH_2PO_4{\cdot}2H_2O$), sodium hydroxide (NaOH), spermine, tetrahydrofuran (THF), triethylamine (TEA), triphosgene, and triphenyl phosphine were purchased from Wako Pure Chemical Industries, Ltd. (Osaka, Japan).

4-Dimethylaminopyridine (DMAP) was purchased from Sigma-Aldrich, Inc. (MO, USA). Di (*t*-butyl) decarbonate ($(Boc)_2O$), 1-hydroxybenzo-triazole hydrate ($HOBt{\cdot}H_2O$), *N, N*-diisopropylethylamine (DIPEA) and benzotriazol-1-yloxy-tri(pyrrolidino)phosphonium hexafluorophosphate (PyBOP) were purchased from Watanabe Chemical Industries, Ltd. (Hiroshima, Japan).

3. Chemical syntheses of thioflavin T derivatives

The synthetic routes of **ThT-OE2** and **ThT-SP** are shown in Scheme S1.

Synthesis of compound A1

To a mixture of 1,4-dioxane (5.5 mL) and water (3 mL), 2-(2-aminoethoxy)ethanol (0.3 mL, 3.0 mmol) was added. Then, 1M NaOH aq. (3 mL) was added dropwise and then $(Boc)_2O$ (720 mg, 3.3 mmol) in 1,4-dioxane (0.5 mL) was added dropwise and stirred at room temperature for 3h. The reaction mixture was evaporated to dryness. The residue was suspended using cold water. The pH was adjusted to about 2 using 5% KHSO4 aq. before it was extracted with ethyl acetate. The organic layer was washed with water and then dried over Na_2SO_4. Filtration followed by solvent evaporation gave compound **A1** as a clear oil (543 mg, 62%); ^{1}H NMR (400 MHz, $CDCl_3$) *δ* 3.75 (2H, q) 3.59–3.54 (4H, m) 3.34 (2H, q) 1.45 (9H, s); ESI-MS (positive ion mode) *m*/*z*, found = 206.1, calculated for $[(M+H)^+]$ = 206.14.

Synthesis of compound A2

NaH in oil (133 mg, 5.5 mmol) was added at 0 °C to a solution of compound **A1** (324 mg, 1.6 mmol) in dry THF (30 mL). Then, bromoacetic acid (0.58 mL) was added dropwise at 0 °C and stirred at 0 °C for 8h. MeOH (15mL) was added to the reaction mixture and the reaction mixture was evaporated to dryness and the residue was suspended with ethyl acetate and water. The organic layer was dried over Na_2SO_4 and filtration followed by solvent evaporation gave compound **A2** (287 mg, 57%); ^{1}H NMR (400 MHz, $CDCl_3$) *δ* 4.17 (4H, s) 3.76 (3H, s) 3.73–3.71 (2H, m) 3.67–3.65 (2H, m) 3.55 (2H, t) 3.33 (2H, q) 1.44 (9H, s); ESI-MS (positive ion mode) *m*/*z*, found = 278.4, calculated for $[(M+H)^+]$ = 278.16.

Synthesis of compound A3

4M NaOH aq. (1.2 mL) was added to a solution of compound **A2** (212 mg, 0.76 mmol) in THF/MeOH (1:1, 8.4 mL). The reaction mixture was stirred at room temperature for 6h before it was evaporated to dryness. The obtained residue was diluted with water and ethyl acetate. The pH of aqueous layer was adjusted to 7 using 1N HCl aq. before the layer was then extracted with ethyl acetate. The organic layer was dried over Na_2SO_4 and filtrated. The filtrate was evaporated to dryness to give compound **A3** (164 mg, 82%); ^{1}H NMR (400 MHz, $CDCl_3$) *δ* 4.17 (2H, s) 3.78–3.76 (2H, m) 3.67–3.65 (2H, m) 3.59 (2H, t) 3.35 (2H, d) 1.45 (9H, s); ESI-MS (positive ion mode) *m*/*z*, found = 264.3, calculated for $[(M+H)^+]$ = 264.14.

Synthesis of compound A4

To a solution of compound ThT-AE (33 mg, 0.11 mmol) in dry DMF (0.3 mL), HOBt·H_2O (21 mg, 0.14 mmol), PyBOP (72 mg, 0.14 mmol) and DIPEA (24 μL, 0.14 mmol) were added. Subsequently, compound **A3** (41 mg, 0.16 mmol) in dry DMF (0.2 mL) was added to the mixture and stirred at room temperature for 3 h. The reaction mixture was then evaporated to dryness and the residue was suspended using CH_2Cl_2. The organic layer was washed with water and then evaporated. The residue was washed with dimethyl ether for 3 times to give compound **A4** as a yellow oil; ^{1}H NMR (400 MHz, $CDCl_3$) *δ* 8.48 (1H, d) 7.84 (2H, d) 7.72 (1H, s) 7.66 (1H, d) 6.90 (2H, d) 4.94 (2H, t) 3.98 (2H, d) 3.93 (2H, s) 3.63 (4H, d) 3.54 (2H,t) 3.31 (2H, s) 3.17 (6H, s) 2.57 (3H, s) 1.42 (9H, s); ESI-MS (positive ion mode) *m*/*z*, found = 557.3, calculated for $[M^+]$ = 557.28.

Synthesis of compound ThT-OE2

The crude compound **A4** (0.11 mmol) was dissolved in dry CH_2Cl_2 (0.75 mL) and TFA (0.15 mL) before the mixture was stirred at room temperature for 7h. The reaction mixture was evaporated and co-evaporated with water to remove TFA. The residue was suspended using CH_2Cl_2, and the organic

layer was washed with water and then evaporated. Finally, the resultant residue was purified using the HPLC system equipped with an octadecyl silica (ODS) gel column to give **ThT-OE2** as a yellow powder (35 mg, 70% from **A3**); ^{1}H NMR (600MHz, Deuterium oxide) δ7.97 (2H, t) 7.73 (3H, t) 6.98 (2H, d) 5.08 (2H, t) 3.68 (2H, t) 3.65 (2H, d) 3.62 (2H, s) 3.59–3.57 (2H, m) 3.42–3.40 (2H, m) 3.15 (2H, t) 3.10 (6H, s) 2.55 (3H, s); ESI-MS (positive ion mode) *m*/*z*, found = 457.2, calculated for [M^+] = 457.23.

Synthesis of compound S1

TFAEE (27 μL, 0.23 mmol) was added dropwise to a solution of spermine (47 mg, 0.23 mmol) in dry MeOH (2 mL) at –78 °C and the mixture was stirred at –78 °C for 30 min. After raising the temperature from –78 °C to 0 °C, $(Boc)_2O$ (304 mg, 1.4 mmol) in dry MeOH (0.2 mL) was added dropwise to the reaction mixture and then stirred at room temperature for 15 h. The pH of reaction mixture was adjusted to 11 using ammonia solution before it was evaporated to dryness. To give compound **S1** (66 mg, 56%), the residue was purified by silica-gel column chromatography with a gradient of 1% to 7% MeOH in CH_2Cl_2; ^{1}H NMR (400MHz, $CDCl_3$) δ3.37 (2H, t) 3.23–3.09 (6H, m) 2.96 (2H, t) 1.92 (2H, t) 1.66 (2H, t) 1.45 (33H, t); ESI-MS (positive ion mode) *m*/*z*, found = 503.5, calculated for [$(M+H)^+$] = 503.38.

Synthesis of compound S2

TEA (9 μL, 64 μmol) and triphosgene (10 mg, 32 μmol) was added to a solution of ThT-AE (9 mg, 32 μmol) in dry CH_2Cl_2 (0.2 mL) and the mixture was stirred at room temperature for 30 min. After the reaction mixture was evaporated to dryness, compound **S1** (17 mg, 32 μmol in dry CH_2Cl_2 (0.3 mL) was added and then stirred at room temperature for 1.5 h. After it was evaporated to dryness, the obtained residue was diluted with CH_2Cl_2. The organic layer was washed with saturated $NaHCO_3$ aq. and then evaporated. The crude compound **S2** as a yellow oil was given by purifying the residue by silica-gel column chromatography with a gradient of 3% to 10 % MeOH in CH_2Cl_2; ESI-MS (positive ion mode) *m*/*z*, found = 840.6, calculated for [M^+] = 840.51.

Synthesis of compound ThT-SP

The crude compound **S2** (32 μmol) was dissolved in MeOH (0.2 mL) and 6N HCl aq. (0.2 mL) before the mixture was stirred at room temperature for 16h. The reaction mixture was evaporated and co-evaporated with water to remove HCl. Finally, the resultant residue was purified using the HPLC system equipped with an octadecyl silica (ODS) gel column to give **ThT-SP** as a yellow powder (1.0 mg, 6% from **S1**); ^{1}H NMR (600MHz, Deuterium oxide) δ7.86 (2H, t) 7.60 (3H, d) 6.87 (2H, d) 4.87 (2H, t) 3.41 (2H, t) 2.99 (6H, s) 2.94–2.90 (4H, m) 2.89 (2H, s) 2.85–2.82 (4H, m) 2.73–2.70 (4H, m) 2.44 (3H, s) 1.78 (1H, s) 1.57 (1H, s) 1.22 (4H, d); ESI-MS (positive ion mode) *m*/*z*, found = 540.7, calculated for [M^+] = 540.35.

Table 1. Fluorescence intensities of ThT, ThT-HE, ThT-AE, ThT-OE2, ThT-SP and ThT-OE11 (3 μM) with oligonucleotides (15 μM) in buffer PBS140KM and PBS153NM.

dye	buffer	Oligonucleotide						
		22AG	26Tel	27Myc	22Kit	20Src	18Ras	dsDNA
ThT	PBS140KM	540 (14)	450 (11)	540 (14)	220 (5.7)	78 (2.0)	290 (7.4)	39 (1)
	PBS153NM	93 (3.6)	130 (4.8)	670 (26)	570 (22)	40 (1.5)	430 (17)	26 (1)
ThT-HE	PBS140KM	130 (16)	240 (29)	440 (53)	140 (17)	54 (6.6)	180 (22)	8.2 (1)
	PBS153NM	15 (3.0)	37 (7.5)	840 (170)	260 (52)	20 (4.0)	310 (62)	4.9 (1)
ThT-AE	PBS140KM	0.96 (8.4)	1.6 (14)	13 (110)	1.7 (15)	4.6 (40)	19 (170)	0.11 (1)
	PBS153NM	0.47 (3.2)	0.41 (2.8)	17 (110)	2.4 (17)	1.7 (12)	26 (180)	0.15 (1)
ThT-OE2	PBS140KM	150 (8.5)	190 (11)	350 (20)	120 (7.1)	75 (4.3)	230 (13)	17 (1)
	PBS153NM	17 (1.9)	43 (4.7)	350 (38)	360 (40)	46 (5.0)	380 (42)	9 (1)
ThT-SP	PBS140KM	350 (6.1)	360 (6.3)	520 (8.9)	240 (4.0)	140 (2.5)	360 (6.2)	58 (1)
	PBS153NM	120 (2.0)	180 (3.0)	530 (8.9)	520 (8.7)	130 (2.2)	550 (9.2)	60 (1)
ThT-OE11	PBS140KM	51 (23)	75 (34)	170 (75)	65 (29)	39 (18)	150 (69)	2.2 (1)
	PBS153NM	4.3 (3.3)	8.1 (6.1)	150 (114)	110 (84)	20 (15)	200 (150)	1.3 (1)

Here, values in parentheses are relative fluorescence intensities obtained by setting fluorescence intensity of a dye with dsDNA to 1. All samples were excited at 415 nm, and the emissions were monitored at 485 nm.

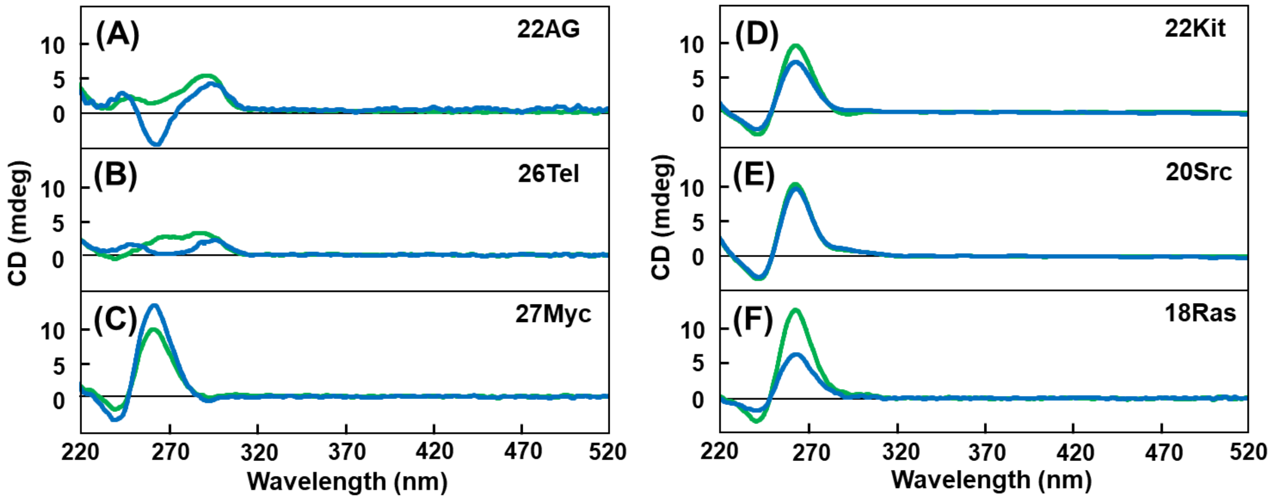

Figure 1. CD spectra for (A) 22AG, (B) 26Tel, (C) 27Myc, (D) 22Kit, (E) 20Src, and (F) 18Ras (12 μM) in PBS150K (green) and PBS153NM (blue). PBS150K (92 mM HPO_4^{2-}, 150 mM K^+, 15 mM Na^+; pH 7.0) was used as a mimic of intracellular ionic components, same as PBS140KM. These data were obtained from reference 17.

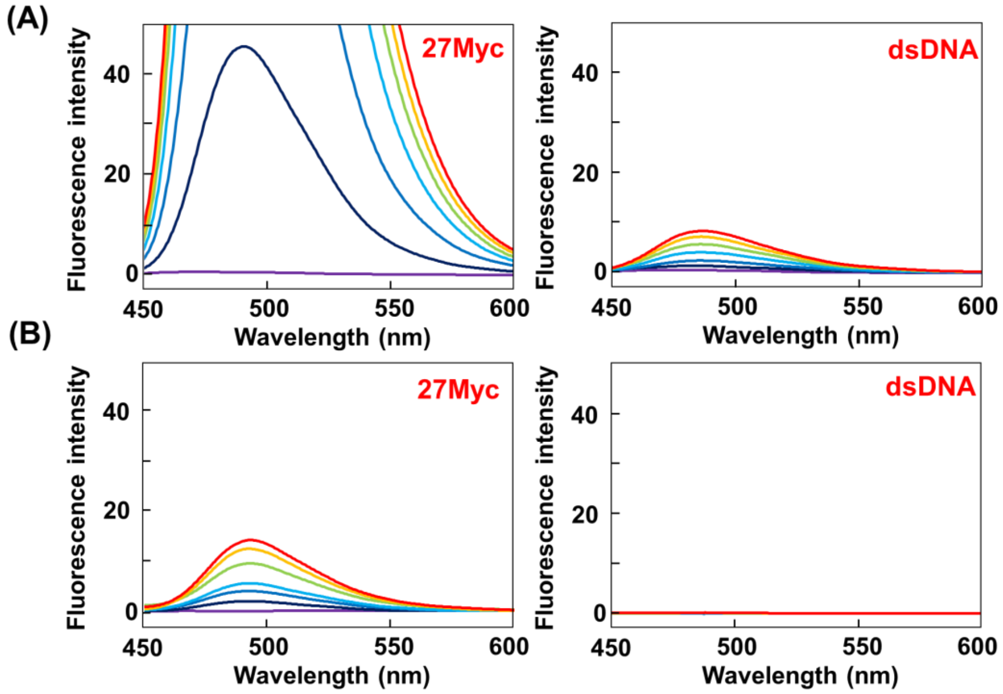

Figure 2. Fluorescence spectra of (A) ThT-HE and (B) ThT-AE (3 μM) in PBS140KM with increasing concentrations of 27Myc (left side) and dsDNA (right side) (0, 1, 3, 6, 9, 12, and 15 μM). Oligonucleotide concentration: 0 μM (purple), 1 μM (navy blue), 3 μM (blue), 6 μM (light blue), 9 μM (green), 12 μM (yellow), and 15 μM (red). These data were enlarged from Figure 4.

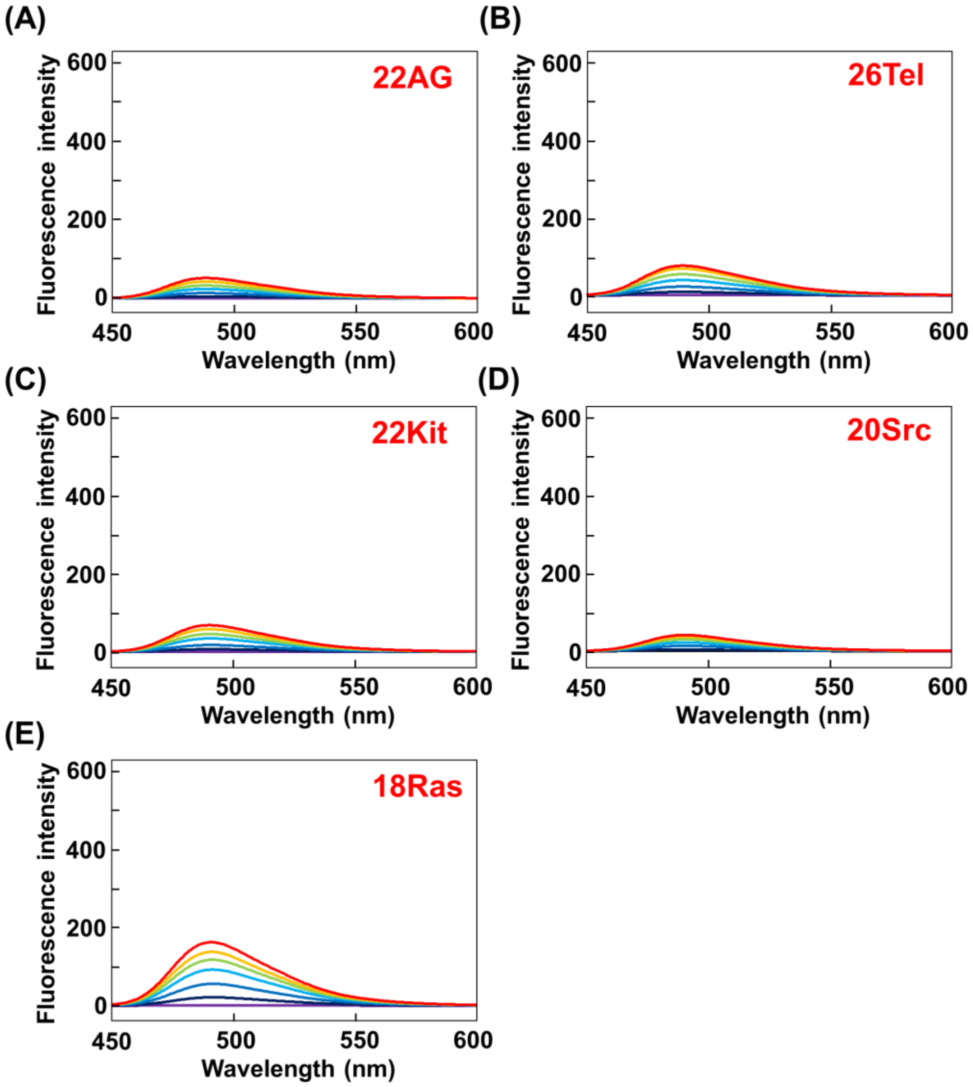

Figure 3. Fluorescence spectra of ThT-OE11 (3 μM) in PBS140KM with increasing concentrations of (A) 22AG, (B) 26Tel, (C) 22Kit, (D) 20Src and (E) 18Ras (0, 1, 3, 6, 9, 12, and 15 μM). Oligonucleotide concentration: 0 μM (purple), 1 μM (navy blue), 3 μM (blue), 6 μM (light blue), 9 μM (green), 12 μM (yellow), and 15 μM (red).

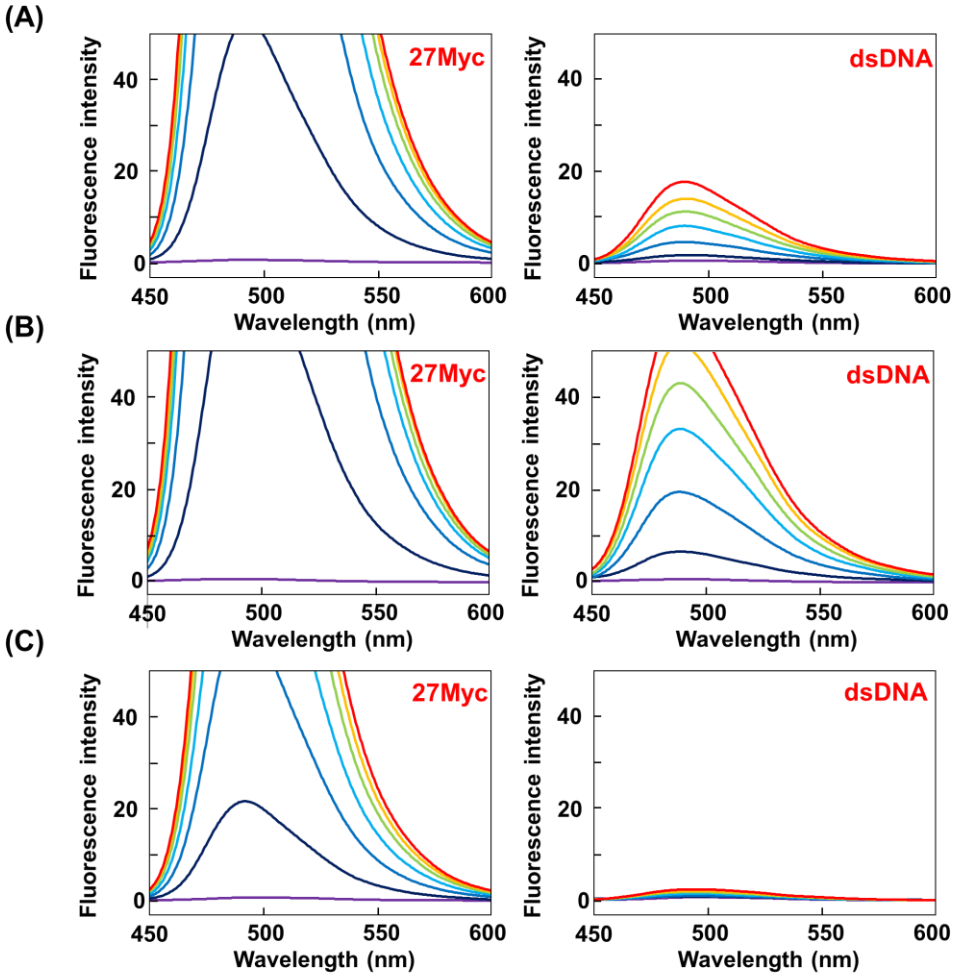

Figure 4. Fluorescence spectra of (A) ThT-OE2, (B) ThT-SP, and (C) ThT-OE11 (3 μM) in PBS140KM with increasing concentrations of 27Myc (left side) and dsDNA (right side) (0, 1, 3, 6, 9, 12, and 15 μM). Oligonucleotide concentration: 0 μM (purple), 1 μM (navy blue), 3 μM (blue), 6 μM (light blue), 9 μM (green), 12 μM (yellow), and 15 μM (red). These data were enlarged from Figure 6.

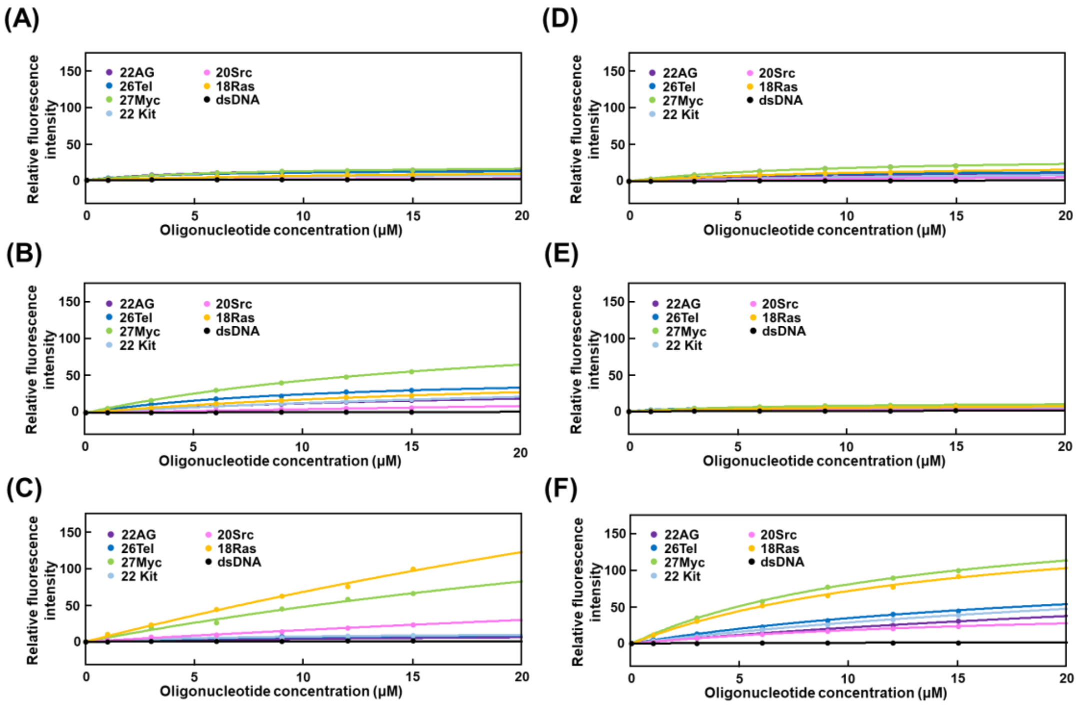

Figure 5. Fluorescence titration curves of (A) ThT, (B) ThT-HE, (C) ThT-AE, (D) ThT-OE2, (E) ThT-SP, and (F) ThT-OE11 (3 μM) in PBS140KM with increasing concentration of oligonucleotides (0, 1, 3, 6, 9, 12, and 15 μM). Oligonucleotides: 22AG (purple), 26Tel (blue), 27Myc (green), 22Kit (light blue), 20Src (pink), 18Ras (yellow), and dsDNA (black).

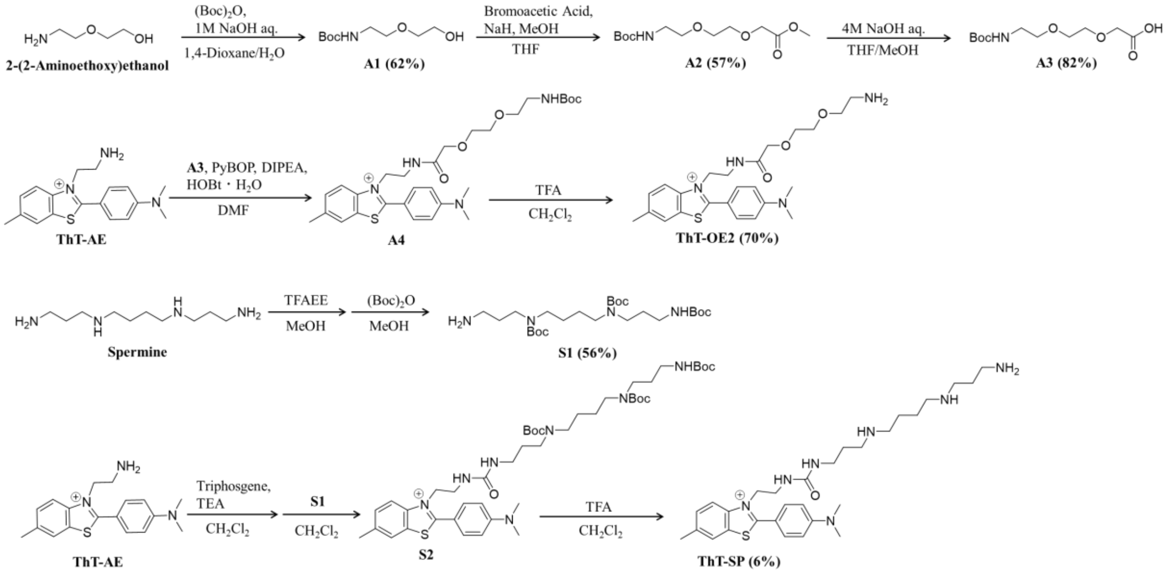

Scheme S1. Synthetic routes for N^3-modified thioflavin T derivatives (ThT-OE2 and ThT-SP).
